# Cost-effectiveness analysis of ultra-hypofractionated radiotherapy and conventionally fractionated radiotherapy for intermediate- to high-risk localized prostate cancer

**DOI:** 10.3389/fonc.2022.841356

**Published:** 2023-01-13

**Authors:** Jiaoxue He, Qingfeng Wang, Qiancheng Hu, Changlin Li

**Affiliations:** ^1^ Department of Clinical Medicine, Southwest Medical University, Luzhou Sichuan, China; ^2^ Department of Oncology, Wenjiang District People’s Hospital, Wenjiang, Chengdu, China; ^3^ Department of Abdominal Oncology, Cancer Center, West China Hospital, Sichuan University, Chengdu, China; ^4^ Department of Oncology, the Seventh People’s Hospital, Chengdu, China

**Keywords:** cost-effectiveness analysis, ultra-hypofractionated radiotherapy, conventionally fractionated radiotherapy, prostate cancer, Markov model

## Abstract

**Background:**

Radiotherapy is an effective curative treatment option for intermediate- to high-risk localized prostate cancer. According to the HYPO-RT-PC trial (ISRCTN45905321), there was no significant difference in 5 years of follow-up in terms of failure-free survival, overall survival, urinary toxicity, and bowel toxicity, while erectile function decreased between ultra-hypofractionated radiotherapy with conventionally fractionated radiotherapy, except that the incidence of urinary toxicity in ultra-hypofractionated radiotherapy was higher at 1 year of follow-up. We evaluated the cost-effectiveness of ultra-hypofractionated radiotherapy and conventionally fractionated radiotherapy for intermediate- to high-risk localized prostate cancer from the Chinese payer’s perspective.

**Methods:**

We developed a Markov model with a 15-year time horizon to compare the cost and effectiveness of ultra-hypofractionated radiotherapy with those of conventionally fractionated radiotherapy for localized intermediate- to high-risk prostate cancer. The outcomes were measured in quality-adjusted life-years (QALYs), incremental cost-effectiveness ratio (ICER), and willingness-to-pay (WTP). Univariable and probability sensitivity analyses were performed to evaluate the robustness of the Markov model.

**Results:**

Based on the Markov model, conventionally fractionated radiotherapy yielded 2.32 QALYs compared with 2.14 QALYs in ultra-hypofractionated radiotherapy in China. The cost of ultra-hypofractionated radiotherapy was found to be decreased by about 14% folds ($4,251.04) in comparison with that of conventionally fractionated radiotherapy. The ICER of conventionally fractionated radiotherapy *versus* that of ultra-hypofractionated radiotherapy was $23,616.89 per QALY in China. The failure-free survival with grade 2 or worse urinary toxicity and the discount rate per annum were the most sensitive parameters utilized in ultra-hypofractionated radiotherapy. The cost-effectiveness acceptability curve showed that conventionally fractionated radiotherapy had 57.7% probability of being cost-effective under the Chinese WTP threshold.

**Conclusion:**

From the perspective of Chinese payers, ultra-hypofractionated radiotherapy was not a cost-effective strategy compared with conventionally fractionated radiotherapy for patients with localized intermediate- to high-risk prostate cancer. Nevertheless, reduction of the grade 2 or worse urinary toxicity of ultra-hypofractionated radiotherapy could alter the results.

## Introduction

Globally, prostate cancer is the second most common malignant tumor affecting millions of middle-aged and elderly men. According to the latest report in 2018, its morbidity ranked second (13.5%), and its mortality ranked fifth (6.7%) (1). In China, the incidence of prostate cancer has increased by more than twofold from 1992 to 2017 (2). About 80% of patients have localized prostate cancer at the time of diagnosis, and about 30%–40% of patients develop distant metastasis and ultimately succumb to the disease within 5 years after the initial diagnosis (1).

Radiotherapy in combination with androgen deprivation therapy is well established as a treatment for intermediate- to high-risk localized prostate cancer (3). One particular area of interest is about which radiotherapy approach is more suitable for intermediate- to high-risk cases. Given that the alpha/beta ratio for prostate cancer is less than 3 Gy, hypofractionated radiotherapy—which has a higher dose per fraction with fewer fractions of radiation—has been intensively studied in prospective clinical trials in localized prostate cancer (4, 5). Hypofractionated radiotherapy ranges from 2.4 to 3 Gy per fraction within 4–6 weeks, resulting in a total dose of 60–70 Gy, while ultra-hypofractionated radiotherapy can reach 35 or 36.25 Gy in 5 fractions over 1 to 2 weeks (6–8). A recent meta-analysis has confirmed that the results in overall survival (HR = 1.12, 95% CI: 0.93–1.35, *p* = 0.219) and prostate cancer-specific survival (HR = 1.29, 95% CI: 0.42–3.95, *p* = 0.661) for hypofractionated radiotherapy were comparable with those for conventionally fractionated radiotherapy (9). Similarly, ultra-hypofractionated radiotherapy, compared with conventionally fractionated radiotherapy, does not improve the 5-year disease-free survival and decrease the late gastrointestinal and genitourinary toxicities in intermediate- and high-risk patients with prostate cancer (10, 11). The cost-effectiveness between ultra-hypofractionated radiotherapy and conventionally fractionated radiotherapy is of utmost importance when determining the best treatment scheme for patients with intermediate- and high-risk localized disease (12).

Recent advances in imaging and treatment planning have made it possible to provide shorter and more convenient schedules at higher doses (13). Several economic analyses of intensity-modulated radiotherapy (IMRT) exist and result in improved outcomes at a lower cost compared with three-dimensional radiation therapy (14–16). With the increasing number of cancer patients, radiotherapy technology has been widely used. However, there are relatively few radiotherapy equipment in developing countries with underdeveloped economy (17). The use of ultra-hypofractionated radiotherapy with shorter treatment courses can reduce travel expenses and increase a patient’s convenience, especially during the COVID-19 pandemic (12, 18).

Given that ultra-hypofractionated radiotherapy provides additional biological benefit, increases a patient’s convenience, and is associated with expensive equipment, the relative economic value of this treatment has received little attention. To address this issue, we have developed a Markov simulation model to evaluate the cost-effectiveness of ultra-hypofractionated radiotherapy compared with conventionally fractionated radiotherapy in patients with intermediate- to high-risk localized prostate cancer from the perspective of a Chinese payer.

## Materials and methods

### Study design of the HYPO-RT-PC trial

HYPO-RT-PC was a multi-national, randomized, open-label, phase III clinical trial with a non-inferiority design (**Table 1**). Patients with intermediate- to high-risk localized prostate cancer received either 42.7 Gy in seven fractions for 2.5 weeks—with an interval of 1 day in the ultra-hypofractionated radiotherapy group—or 78 Gy at 2 Gy/fraction for 5 days per week over an 8-week period in the conventional fractionated radiotherapy group. The patients were permitted to receive androgen deprivation therapy in two groups. The 120 (20%) patients and 118 (20%) patients in the ultra-hypofractionated radiotherapy group and conventional fractionated radiotherapy group received volumetric-modulated arc therapy or intensity-modulated radiotherapy, respectively. All patients in the two groups received image-guided radiotherapy (IGRT). In the HYPO-RT-PC trial, the proportion and the duration of treatment regimens used in the second-line and the third-line metastatic prostate cancer treatments were not applied (10, 19).

**Table 1:** Baseline demographics, clinical characteristics, and radiotherapy details were recorded between CRT and UHRT in the HYPO-RT-PC trial.

Characteristics	CRT (*n* = 591)	UHRT (*n* = 589)	*P*-value
Age (years, range)	69 (65–72)	68 (64–72)	
Intermediate risk (*n*, %)	527 (89%)	527 (89%)	
High risk (*n*, %)	64 (11%)	62 (11%)	
3DCRT (*n*, %)	471 (79.7%)	471 (80%)	
VMAT/IMRT (*n*, %)	120 (20.3%)	118 (20%)	
BED (Gy)	130	129.52	
Total radiotherapy dose (Gy)	78	42.7	
Frequency of radiotherapy (f)	39	7	
Single dose of radiation (Gy)	2	6.1	
Total time of radiotherapy (days, range)	57 (55–59)	16 (15–17)	
5-year failure-free survival rate	84%	84%	0.99
5-year overall survival rate	96%	94%	0.62
Urinary toxicity (≥grade 2)	2%	6%	0.0037

3DCRT, three-dimensional conformal radiotherapy; VMAT/IMRT, volumetric-modulated arc therapy or intensity-modulated radiotherapy; CRT, conventionally fractionated radiotherapy; UHRT, ultra-hypofractionated radiotherapy; BED, biological effective dose: the calculation formula is D [1 + d/(α/β)], where D is total radiotherapy dose, and d is a single dose of radiation. The value of α/β is 3 Gy.

### Markov model

**Figure 1:**
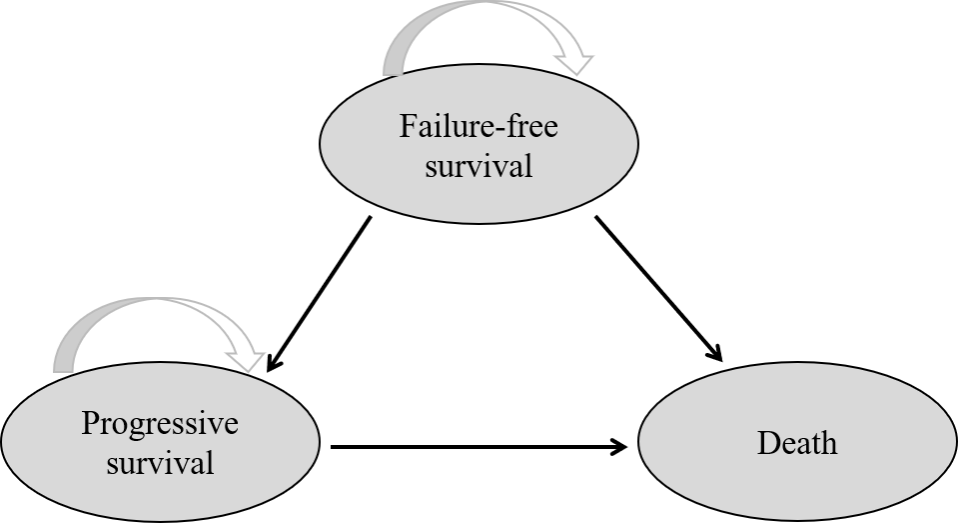
Network of three health states. The arrow indicates from one state into another or staying in the original state.

**Figure 2:**
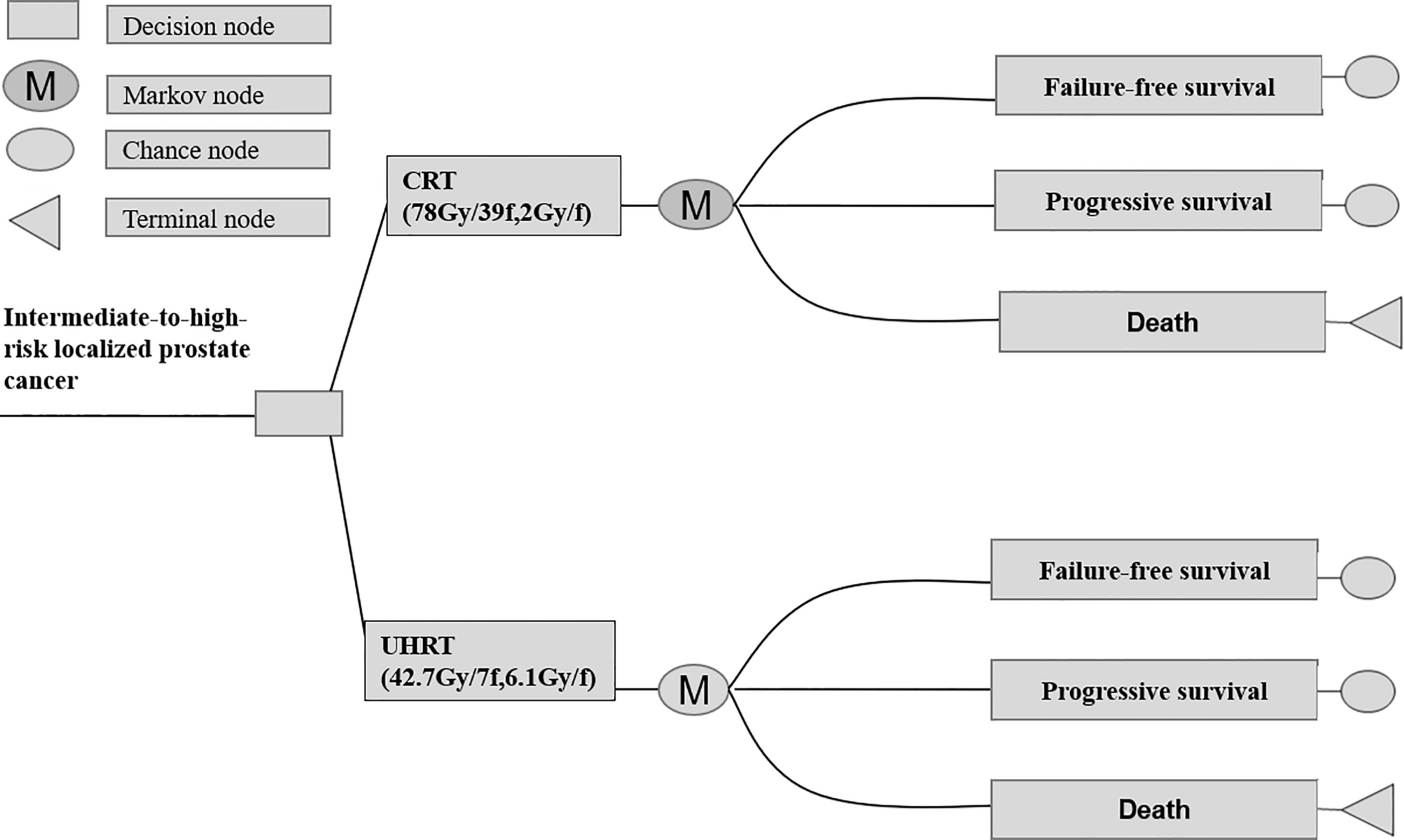
Abbreviated decision tree and Markov model used to compare CRT and UHRT for intermediate- to high-risk localized prostate cancer. CRT, conventionally fractionated radiotherapy; UHRT, ultra-hypofractionated radiotherapy.

According to the HYPO-RT-PC trial (ISRCTN45905321) protocol, a Markov model programmed in TreeAge Pro software 2011 (TreeAge Software LLC, Williamstown, MA, USA) was used for comparing the economic consequences and therapeutic efficacy of ultra-hypofractionated radiotherapy from the Chinese payer’s perspective (10). Three states were included—failure-free survival (FFS), progressive survival (PS), and death (**Figure 1**). Moreover, a time period of 15 years was used, *i*.*e*., almost all patients were assumed in the model to live for less than 15 years. The average healthy life expectancy reached 83 years with a 15-year time horizon in our study, which was more than the estimated life expectancy of age 60 years in men in China according to the World Health Organization (WHO) reports (20). All patients started in the FFS state, and then they could progress to either the PS or death state based on transition probabilities. The PS state could not enter the FFS state, as death was an absorbing state (**Figure 2**). In the HYPO-RT-PC trial, there were only 5 years of FFS and overall survival after diagnosis; thus, the survival rate data of 5–15 years were obtained from previously published papers (21). The Kaplan–Meier survival data presented graphically were extracted from survival curves using WebPlot-Digitizer (http://apps.automeris.io/wpd/index.zh_CN.html, which were further used to fit parametric survival models (22). The survival models of two groups were fitted with Weibull distribution function. The transition probabilities between health states in the model were derived from published literature, and prospective utility measurement was preferred whenever possible. The transition probability from FFS to death was 0.0003 of Sweden’s all-cause death probability (23), of which the FFS to PS and PS to death in each cycle were estimated by the following formula: *P* (*t* → *t* + 1) = -exp[λ(*t*)^γ - λ(*t* + 1) ^γ)], where *t* stood for the current cycle number in the Markov model (24).

### Utility and cost

The quality-adjusted life year (QALY) is an indicator composed of the length and the quality of life, calculated as the product of a utility value from 0 for death to 1 for perfect health (25). The Quality of Life 5D-5L (EQ-5D-5L) instrument was used to measure the health-related quality of life (26). Data on the utilities of different health states in patients with prostate cancer were collected from previous publications (**Table 2**). From the perspective of a Chinese society, our study took into account the direct medical costs, including radiotherapy, urinary toxicity, digital rectal examination, blood test, imaging examination, hospitalization, androgen deprivation therapy, chemotherapy, and supportive treatment costs (**Table 3**). We assumed that all patients received a total of 24 months of adjuvant androgen deprivation therapy based on the National Comprehensive Cancer Network (NCCN) practice guideline for prostate cancer (32), of which the costs were obtained from national price announcement in the third-grade first-class hospitals in Chengdu, China, and the direct non-medical costs only took into account the transportation costs. We did not consider the indirect labor costs due to the average age of the two groups being more than 60 years, which is the official retirement age in China (33).

**Table 2:** Summary of model parameters and assumptions.

Parameter	Health utility value	References	Distribution
	Mean (range)		
Utility of biochemical recurrence	0.74 (0.592–0.888)	(27, 28)	β
Utility of clinical metastasis	0.25 (0.2–0.3)	(27, 28)	β
U_CRT_UT	0.91 (0.7274–1)	(27, 28)	β
U_UHRT_UT	0.85 (0.7265–1)	(28)	β
U_PS	0.61 (0.49–0.73)	(10, 27, 28)	β
Discount rate (%)	3 (0–8)	(25)	β

U_CRT_UT, utility of conventionally fractionated radiotherapy with grade 2 or worse urinary toxicity; U_UHRT_UT, ultra-hypofractionated radiotherapy with grade 2 or worse urinary toxicity; U_PS, utility of progressive survival, which was calculated according to the weight of biochemical recurrence and clinical metastasis.

Utility was drawn from the β distribution.

**Table 3:** Key cost parameters and related assumptions.

Unit cost ($)	CRT	UHRT	References	Distribution
	Mean (range)	Mean (range)		
Radiation oncologist	2.17 (1.74–2.61)	2.17 (1.74–2.61)	(29)	γ
Pelvic enhanced CT	83.94 (67.16–100.73)	83.94 (67.16–100.73)	(29)	γ
Mask design and production	13.92 (11.13–16.70)	13.92 (11.13–16.70)	(29)	γ
Body membrane	78.29 (62.63–93.95)	78.29 (62.63–93.95)	(29)	γ
Body frame	5.22 (4.18–6.26)	5.22 (4.18–6.26)	(29)	γ
Real-time radiotherapy monitoring	7.25 (5.80–8.70)	7.25 (5.80–8.70)	(29)	γ
Complex analog positioning of special X-ray machine	135.70 (108.56–162.84)	135.70 (108.56–162.84)	(29)	γ
Specific computer treatment planning system	316.06 (252.85–379.27)	316.06 (252.85–379.27)	(29)	γ
Intensity-modulated radiation therapy	173.98 (139.18–208.77)	173.98 (139.18-208.77)	(29)	γ
X knife therapy (first time)		724.91 (579.93–869.89)	(29)	γ
X knife therapy		362.46 (289.96–434.95)	(29)	γ
Image-guided radiotherapy (first time)		195.15 (156.12–234.18)	(29)	γ
Image-guided radiotherapy		160.64 (128.51–192.77)	(29)	γ
Routine blood test	2.75 (2.20–3.31)	2.75 (2.20–3.31)	(29)	γ
Biochemistry blood test	14.50 (11.60–17.40)	14.50 (11.60–17.40)	(29)	γ
Electrocardiogram	4.93 (3.94–5.92)	4.93 (3.94–5.92)	(29)	γ
Transportation cost	1.45 (1.16–1.74)	1.45 (1.16–1.74)	Local estimate	γ
Hospitalization fees/day	10.87 (8.70–13.05)	10.87 (8.70–13.05)	(29)	
Upper abdominal plain + pelvic enhanced MRI	310.99 (248.79–373.18)	310.99 (248.79–373.18)	(29)	γ
Head plain CT	72.49 (58.00–86.99)	72.49 (58.00–86.99)	(29)	γ
Bone scan	145 (116–174)	145 (116–174)		
Digital rectal examination	2.17 (1.74–2.61)	2.17 (1.74–2.61)	(29)	γ
PSA	14.21 (11.37–17.05)	14.21 (11.37–17.05)	(29)	γ
Goserelin (month)	396.67 (317.33–476.01)	396.67 (317.34–476.01)	(29)	γ
Bicalutamide (month)	72.49 (57.99–86.99)	72.49 (57.99–86.99)	(29)	γ
Docetaxel (month)	644.94 (515.95–773.93)	644.94 (515.95–773.93)	(29, 30)	γ
Abitrone (month)	579.61 (463.69–695.53)	579.61 (463.69–695.53)	(29, 30)	γ
Kabatasai (month)	5,617.80 (4,494.23–6,741.35)	5,617.79 (4,494.23–6,741.35)	(29, 30)	γ
Supportive treatment (month)	543.70 (434.96–652.45)	543.70 (434.96–652.45)	(31)	γ
Urinary toxicity	960 (768–1,152)	960 (768–1,152)	(27)	Γ

PSA, prostate cancer-specific antigen; CRT, conventionally fractionated radiotherapy; UHRT, ultra-hypofractionated radiotherapy.

Costs were drawn from the γ distribution.

### Cost-effectiveness analysis

All costs were presented in 2020 US dollar, and future costs and health outcomes were discounted to the current year with an annual rate of 3%, reflecting the average annual inflation rate in China (34). Clinical effectiveness was expressed in QALYs, which was calculated as the sum of the product of health utilities weight in a given state and the number of life years gained (35). The cost-effectiveness analysis was evaluated using incremental cost-effectiveness ratios (ICERs) between ultra-hypofractionated radiotherapy and conventionally fractionated radiotherapy (25, 36). The willingness-to-pay (WTP) threshold value for cost-effective analysis was three times the gross domestic product per capita of China in 2020, which was set at $31,510 per QALY according to the WHO guidelines (37).

### Sensitivity analysis

The robustness of our model parameters was estimated by one-way sensitivity analysis and probability sensitivity analysis. A series of deterministic sensitivity analyses was performed to test the robustness of base case results, and the parameters were obtained by varying the base case by 20% in the deterministic sensitivity analysis (38). We assumed a beta probability distribution for the health utility values and a gamma distribution for cost parameters, respectively (**Tables 2**, **3**). Moreover, the discount rate considered as β distribution was varied (0%–8%) within the sensitivity analysis (**Table 2**). The one-way sensitivity analysis results were demonstrated as a tornado diagram with the most influential model parameters. We performed probabilistic sensitivity analyses with 1,000 Monte Carlo simulations, with all of the input variables varied simultaneously with a specific pattern of distribution. Lastly, a second-order Monte Carlo simulation was developed to estimate the expected values of costs and effectiveness in the base case (39).

## Results

### Base case results

**Figure 3:**
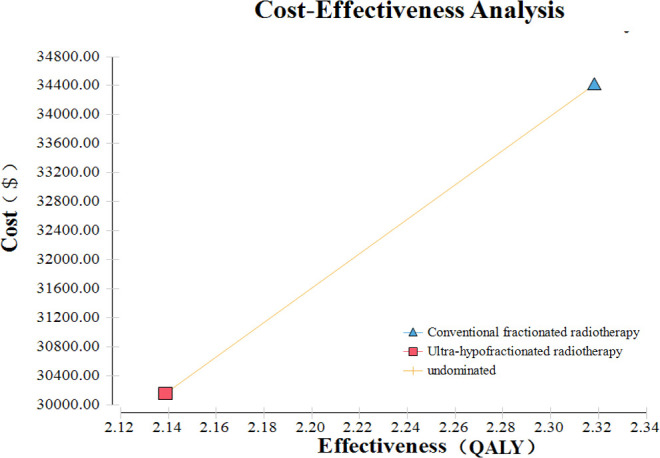
Cost-effectiveness analysis of ultra-hypofractionated radiotherapy and conventionally fractionated radiotherapy for intermediate- to high-risk localized prostate cancer.

Based on the Markov model, conventionally fractionated radiotherapy yielded 2.32 QALYs compared with 2.14 QALYs of ultra-hypofractionated radiotherapy in China (**Figure 3**). Treatment with conventionally fractionated radiotherapy costs $34,411.85 compared with $30,160.81 for ultra-hypofractionated radiotherapy. The cost of ultra-hypofractionated radiotherapy was found to be decreased by about 14% folds ($4,251.04) in comparison with that of conventionally fractionated radiotherapy. The ICER of conventionally fractionated radiotherapy *versus* that of ultra-hypofractionated radiotherapy was $23,616.89 per QALY in China. The details are listed in **Table 4**.

**Table 4:** Cost-effectiveness analysis of CRT and UHRT.

	CRT	UHRT
Effectiveness (QALYs)	2.32	2.14
Cost ($)	34,411.85	30,160.81
Incremental effectiveness (QALYs)	0.18	/
Incremental cost ($)	4,251.04	/
Incremental cost/effectiveness ($/QALY)	23,616.89	/
Average cost/effectiveness ($/QALY)	14,843.97	14,102.60

CRT, conventionally fractionated radiotherapy; UHRT, ultra-hypofractionated radiotherapy.

/, no data.

### Sensitivity analysis

**Figure 4:**
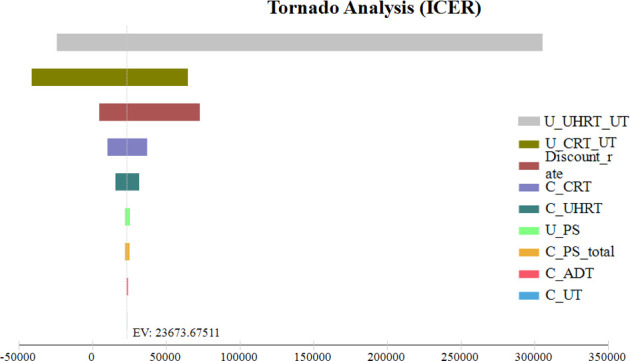
One-way sensitivity analysis. This diagram shows the incremental cost effectiveness ratio of CRT for different model input parameters from the perspective of a Chinese society. U_UHRT_UT, utility of ultra-hypofractionated radiotherapy with grade 2 or worse urinary toxicity; U_CRT_UT, utility of conventionally fractionated radiotherapy with grade 2 or worse urinary toxicity; C_CRT, cost of conventionally fractionated radiotherapy; C_UHRT, cost of ultra-hypofractionated radiotherapy; U_PS, utility of progressive survival; C_PS_total, total cost of progressive survival; C_ADT, cost of androgen deprivation therapy; C_UT, cost of grade 2 or worse urinary toxicity.

The results of the one-way sensitivity analysis of our Markov model are presented in **Figure 4**. The most sensitive parameters were the ultra-hypofractionated radiotherapy utility of FFS with grade 2 or worse urinary toxicity and the discount rate per annum. When the utility of FFS with grade 2 or worse urinary toxicity of ultra-hypofractionated radiotherapy varied from 0.72 to 0.77, the ICER of conventionally fractionated radiotherapy *versus* that of ultra-hypofractionated radiotherapy ranged from $32,615.86 to $5,850,488.91 per QALY, which exceeded the WTP threshold of $31,510 per QALY. When the conventionally fractionated radiotherapy utility of FFS with grade 2 or worse urinary toxicity was 0.73 and 0.82 QALY, the effectiveness of ultra-hypofractionated radiotherapy was higher than that of conventionally fractionated radiotherapy, with an increase of 0.12 and 0.04 QALY, respectively. The cost of ultra-hypofractionated radiotherapy was $4,251.04 less than that of conventionally fractionated radiotherapy, while its QALY was higher. Therefore, ultra-hypofractionated radiotherapy had an absolute cost-effectiveness advantage. In addition, conventionally fractionated radiotherapy was no longer cost-effective when the discount rate per annum achieved was 3.68% or more.

**Figure 5:**
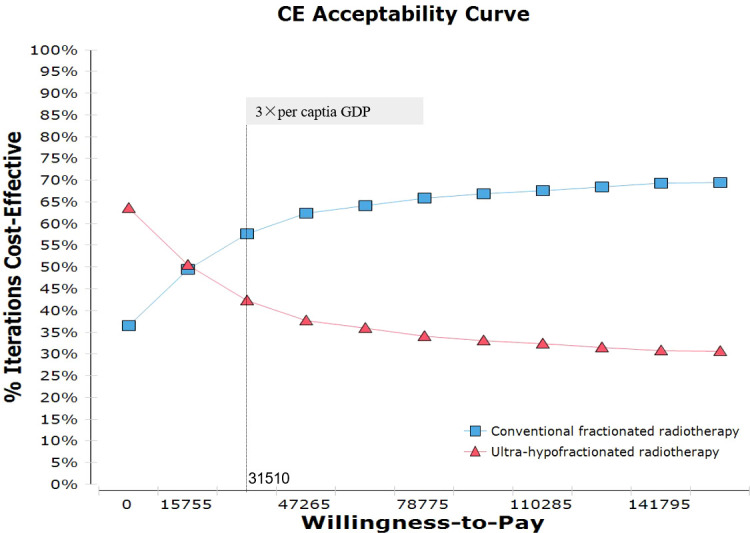
Probabilistic sensitivity analysis for cost-effectiveness of treatment strategies for CRT and UHRT for intermediate- to high-risk localized prostate cancer. The dotted vertical lines represent the willingness-to-pay thresholds ($) from the payer’s perspective of a Chinese society. CRT, conventionally fractionated radiotherapy; UHRT, ultra-hypofractionated radiotherapy.

**Figure 5** illustrated the cost-effectiveness acceptability curve associated with the proportion of the intervention at any threshold value of WTP, showing that conventionally fractionated radiotherapy had 57.7% probability of being cost-effective at the Chinese WTP threshold. When the hypothetical WTP threshold increased to $141,795, the probability for conventionally fractionated radiotherapy to be cost-effective was 69.3%.

## Discussion

Our study demonstrated that conventionally fractionated radiotherapy yielded an additional 0.18 QALYs than ultra-hypofractionated radiotherapy, leading to an ICER of $23,616.89 per QALY in China. Although the unit cost of ultra-hypofractionated radiotherapy was found to be decreased by about 14% folds ($4,251.04) in comparison with that of conventionally fractionated radiotherapy, from the perspective of Chinese payers, it was not a cost-effective strategy in patients with localized intermediate- to high-risk prostate cancer.

In recent years, costs were derived from the continuous advancement of technology and the upgrading of radiotherapy-relevant devices. Compared with tridimensional radiotherapy, the incremental cost of IMRT for prostate cancer was $5,553.78 in the Brazilian health system (40). Given no differences in the cost of radiotherapy-related devices in the HYPO-RT-PC trial, the cost of ultra-hypofractionated radiotherapy was lower than that of conventionally fractionated radiotherapy in our study. In a previously published cost-effectiveness analysis, stereotactic body radiotherapy (SBRT) that consisted of a total dose of 37 Gy over five fractions was the most cost-effective radiation treatment modality for patients with intermediate-risk prostate cancer (41). However, SBRT with better long-term outcomes is a prerequisite for a highly accessible and more cost-effective intervention. Actually, the phase III HYPO-RT-PC trial—the first randomized controlled trial comparing ultra-hypofractionated with conventional fractionation—confirmed that there was no statistical difference in FFS (84 *vs*. 84%, *p* = 0.99) between the two groups with localized intermediate- to high-risk prostate cancer. However, ultra-hypofractionated radiotherapy resulted in higher genitourinary toxicity in grade 2 or worse (10).

The optimal utility of FFS for ultra-hypofractionated radiotherapy remains to be determined, and its cost-effectiveness is strongly related to the cost of grade 2 or worse urinary toxicity. In China, the utility of prostate cancer- and treatment-related health status in patients with ultra-hypofractionated radiotherapy was rarely reported, so we obtained the utility values from previously published studies (27, 28, 42). The most sensitive parameter was the ultra-hypofractionated radiotherapy utility of FFS with grade 2 or worse urinary toxicity in the tornado diagrams. The results of a one-way sensitivity analysis revealed that conventionally fractionated radiotherapy was not a cost-effective strategy in patients with localized intermediate- to high-risk prostate cancer when the utility of FFS with grade 2 or worse urinary toxicity of ultra-hypofractionated radiotherapy varied from 0.72 to 0.77. However, in previously published cost-effectiveness analyses, the utility of FFS for symptoms occurring with treatment varied from 0.71 to 0.89, being likely to have substantially altered the results of ICER (28). Most patients did not receive the combination of IMRT and IGRT in the HYPO-RT-PC trial, which has been widely used for ultra-hypofractionated radiotherapy in China. Therefore, the utility of prostate cancer- and treatment-related health states in China was more urgently needed for cost-effectiveness analysis in the future.

Due to severe urinary toxicity, ultra-hypofractionated radiotherapy has a lower health utility and relatively no cost-effective advantage. Many studies have analyzed and compared the cost-effectiveness of different prostate radiotherapy modalities. In a cost-effectiveness analysis of IMRT and 3D-CRT for localized prostate cancer, IMRT was more cost-effective than 3D-CRT, with an increase of 0.023 QALYs and ICER (incremental cost–benefit ratio) of $26,768/QALY (15). Moreover, in the cost-effectiveness study of three-dimensional radiotherapy, intensity-modulated radiotherapy, and hypofractionated radiotherapy, the cost per QALY was €7,160, €6,831, and €6,019, respectively, and the QALYs obtained were 5.753, 5.956, and 5.957 QALYs, respectively. Hypofractionated radiotherapy was more cost-effective with a lower cost and higher QALYs (31). However, one study had provided the opposite conclusion that SBRT was associated with higher adverse reactions, obtaining 0.03 QALYs lower than IMRT, which is relatively not cost-effective unless the willingness-to-pay threshold is less than $100,000 (43). The results from our Markov model also indicated that ultra-hypofractionated radiotherapy was an economical treatment option only when the WTP was less than $21,522 due to its higher urinary toxicity and lower QALY. With the progress of science and technology and the innovation of radiotherapy technology, the adverse effects of hypofractionated or ultra-hypofractionated radiotherapy have been well controlled, which will become a cost-effective treatment scheme compared with conventional radiotherapy.

Some limitations of the present study are subject to further discussion. First, the limitations in our study were raised primarily from the quality of the inputs used to inform the Markov model. We did not have access to utility and transition probability from a real-world study in China. We acquired transition probability by simulating the survival curves, a method adopted by other similar cost-effectiveness studies (44). Second, due to the lack of long-term outcomes in China, we obtained primary prognostic data of interest mainly from patients in Sweden and Denmark. Third, the difference in late toxicity between ultra-hypofractionated radiotherapy and conventionally fractionated radiotherapy was not considered in the present study. Lastly, some other factors, such as the time away from home, education, and religion, would influence the choice of treatment protocol for patients with localized intermediate- to high-risk prostate cancer.

In conclusion, compared with conventionally fractionated radiotherapy, ultra-hypofractionated radiotherapy is not a cost-effective strategy for patients with localized intermediate- to high-risk prostate cancer from the perspective of Chinese payers. However, reduction of the grade 2 or worse urinary toxicity of ultra-hypofractionated radiotherapy may alter the outcomes.

## Data availability statement

The original contributions presented in the study are included in the article/supplementary material. Further inquiries can be directed to the corresponding author.

## Author contributions

Conceptualization: JH and CL. Methodology: JH and QW. Writing of the original draft: JH, QH, and CL. All authors contributed to the article and approved the submitted version.
